# Supplementary material for: Echocardiographic Patterns of Left Ventricular Diastolic Function in Cardiac Amyloidosis: An Updated Evaluation
Source: J Clin Med. 2021 Oct 23;10(21):4888. doi: 10.3390/jcm10214888 (PMC8584963; doi:10.3390/jcm10214888)
Supplement: Supplementary file 1 [file jcm-10-04888-s001.zip › jcm-1411127-supplementary.pdf]

Table S1: Characteristics of 464 patients according to cardiac amyloidosis type

Characteristics	MV	All n=464	AL n=157	ATTRv n=131	ATTRwt n=176	p
Age, years	0	75(65;81)	66(57;74)	71(63;77)	82(77;86)	**0.0001**
Men	0	327(70)	102(65)	85(65)	140(80)	**0.004**
BMI, kg/m^2	2	24(22;27)	24(22;26)	24(22;26)	25(23;27)	**0.03**
Atrial Fibrillation	0	85(18,3)	12(7.6)	15(11.5)	66(37.5)	**<0.001**
NYHA class III-IV vs I-II	16	21(47.9)	80(54.4)	54(41.9)	81(47.0)	0.11
SBP, mmHg	22	121(107;135)	114(102;130)	121(110;131)	126(111;143)	**0.0001**
HR, beats/min	2	74(65;84)	79(71;90)	72(62;79)	71(62;80)	**0.0001**
NT-proBNP, ng/l	12	3593(1685;7168)	4056(2263;12132)	2511(673;5410)	3840(2146;6418)	**0.0001**
Troponin T us, ng/l	53	72(44;108)	86(51;141)	57(30;87)	71(45;96)	**0.0001**
Creatinine, µmol/l	4	108(85;140)	103(79;150)	100(80;133)	112(92;143)	**0.02**
Sodium level, mmol/l	5	139(137;141)	138(135;141)	140(138;142)	140(138;142)	**0.0002**
Mayo Clinic Stage*	18					
I			5(4)	NA	NA	
II			17(12)			
III			117(84)			
TTR Stage**	7					
I			NA	72(56,3)	54(31)	**<0.001**
II				32(25)	72(42)	
III				24(18,7)	46(27)	
PR, ms***	86	190(160;220)	180(160;200)	186(160;220)	200(180;226)	**0.003**
QRS, ms	78	100(80;124)	90(80;110)	100(86;128)	120(96;140)	**0.0001**
Bundle branch block	46	153(37)	30(21)	40(34)	83(53)	**<0.001**
Low voltage	154	172(55)	93(79)	34(43)	45(40)	**<0.001**
LAVI, mL/m^2	30	46 (37-59)	40 (33-51)	44 (36-58)	51 (44-63)	**0.0001**

Values are median (IQR), or n(%). X^2 or Kruskal-Wallis'test.

AL: light-chain amyloidosis; BMI: body mass index ; HR: heart rate; ATTRv: hereditary transthyretin amyloidosis; LAVI : Left Atrial Volume index ; MV: missing values; NYHA: New York Heart Association; SBP: systolic blood pressure; ATTRwt: wild-type transthyretin amyloidosis.

*from (9), **from (10), ***for patients in sinus rhythm (n=363)

Table S2: Baseline echocardiographic data according to cardiac amyloidosis type

	MV	All n=464	AL n=157	ATTRv n=131	ATTRwt n=176	p
IVST, mm	0	17(15;20)	15(14;18)	18(15;20)	18(16;21)	**0.0001**
LVM index, g/m^2	3	170(135;215)	141(119;172)	191(151;229)	184(155;227)	**0.0001**
GLS, -%	13	10(8;14)	10(8;14)	10(8;14)	10(7;13)	0.87
LVEF, %	0	50(40;60)	57(45;63)	49(36;60)	47(38;57)	**0.0001**
Mean S'm velocity, cm/s	37	4.5(3.5;6)	5.5(4;7.5)	4.5(3.5;6)	4(3.5;5)	**0.0001**
Cardiac index, l/min/m^2	7	2.25(1.7;2.8)	2.5(2.0;3.0)	2.2(1.6;2.7)	2.1(1.7;2.7)	**0.009**
LAV index, ml/m^2	30	46(37;59)	40(33;51)	44(36;58)	51(44;63)	**0.0001**
E_m, cm/sec	4	84(69;101)	87(68;105)	83(62;97)	84(73;101)	0.06
E_m deceleration time, ms	5	159(123;205)	157(120;212)	158(124;216)	160(129;198)	0.75
E_m/A_m ratio*	5	1.8(0.9;3.0)	1.6(0.8;3.0)	1.8(0.8;3.0)	2.0(1.1;2.9)	0.30
E_m/e' ratio**	11	17(13;24)	17(12;25)	17(12;22)	18(13;24)	0.52
Septal e' velocity, cm/s	34	4(3;5)	5(4;6)	4(3;5)	4(3;5)	**0.004**
Lateral e' velocity, cm/s	9	6(4;7)	5(4;7)	6(4;7)	6(4;8)	0.64
TR velocity, cm/s***	59	269(236;297)	271(233;290)	265(232;299)	272(243;303)	0.29
TAPSE, mm	8	15(11;19)	16(12;20)	15(11;19)	14(10;18)	**0.02**
S't' velocity, cm/s	11	10(8;12)	11(9;13)	9(8;12)	9(7;11)	**0.0001**

Values are median (IQR). Same abbreviations as Table 1 and E/e': mitral early diastolic peak-flow velocity/early diastolic velocity by TDI ratio; GLS: global longitudinal strain; IVST: Interventricular septum thickness; LAVindex: left atrial volume index; LVEF: left ventricular ejection fraction; LVM : left ventricular mass; S'm : mean systolic doppler tissue velocity at mitral annulus; S't : mean systolic doppler tissue velocity at tricuspid annulus; TAPSE: tricuspid annular plane systolic excursion; TR: tricuspid regurgitation.

X^2 or Kruskal-Wallis'test.

*for patients in sinus rhythm and available E/A ratio (n=358), **average E/e', except for n=26 patients (calculated on lateral e' only), ***only for patients with TR

Table S3: Univariate and multivariate analyses for elevated LV filling pressure (Grade III+II versus I).

Characteristics	OR	CI	P univariate	OR	CI	P multivariate
Age, years	1.01(per year)	0.99-1.03	0.16			
Men	0.95	0.6-1.5	0.83			
Amyloidosis type						
AL	1(ref)	-	-			
ATTRv	1.1	0.63-1.85	0.78			
ATTRwt	1.5	0.9-2.5	0.11			
Atrial Fibrillation	1.2	0.7-2.0	0.53			
SBP, mmHg	0.99(per mmHg)	0.98-1.0	0.87			
eGFR< 45 ml/min/1.73m^2	1.3	0.8-2.1	0.37			
NYHA class, III-IV vs I-II	2.7	1.7-4.2	<0.001	1.9	1.1-3.2	**0.01**
LVEF, %	0.96(per %)	0.94-0.97	<0.001			
Mean mitral S' velocity, cm/s	0.54(per cm/sec)	0.47-0.63	<0.001			
IVST, mm	1.2(per mm)	1.1-1.3	<0.001	1.1	1.1-1.2	**0.001**
LVMi >170g/m^2 (median)	2.5	1.6-3.9	<0.001			
GLS, -%	0.9(per unit)	0.86-0.95	<0.001	0.9	0.89-0.99	**0.03**
NT-proBNP>3600ng/l (median)	3.3	2.1-5.2	<0.001	2.3	1.4-3.9	**0.002**
Troponin T us>72ng/l (median)	3.1	1.9-5.0	<0.001			
Bundle branch block	1.7	1.0-2.7	0.048			

Same legends as Tables 1 and 2 Supp

Table S4a: Predictors of diastolic function (Grade III+II vs I) by univariates analyses depending on amyloidosis type

Characteristics	AL			ATTRv			ATTRwt		
	OR	CI	P	OR	CI	P	OR	CI	P
Age, years	0.98	0.95;1.02	0.32	1.1	1.0;1.1	0.03	1.0	0.9;1.1	0.84
Men gender	0.9	0.4;1.9	0.77	0.5	0.2;1.1	0.09	1.7	0.7;4.0	0.21
Atrial Fibrillation	2.4	0.5;11.5	0.26	6.8	0.9;53.9	0.06	0.5	0.2;1.0	0.07
SBP, mmHg	0.99	0.97;1.0	0.12	1.0	0.9;1.0	0.84	1.0	0.98;1.0	0.72
eGFR<45 ml/min/1.73m2	1.3	0.6;3.2	0.52	3.8	1.1;13.7	0.04	0.6	0.3;1.4	0.26
NYHA class, III-IV vs I-II	5.9	2.6;13.6	<0.001	4.0	1.5;10.1	0.002	1.1	0.5;2.2	0.87
LVEF, %	0.96	0.92;0.99	0.007	0.9	0.9;0.96	<0.0001	0.99	0.96;1.0	0.32
IVST, mm	1.2	1.0;1.4	0.01	1.3	1.1;1.5	0.0001	1.1	1.0;1.3	0.03
LVMi>170 (median)	2.9	1.2;7.3	0.02	5.1	2.2;12.2	<0.001	1.3	0.6;2.7	0.48
LGS, -%	0.89	0.81;0.97	0.005	0.9	0.8;0.98	0.01	0.9	0.8;1.0	0.06
NT-proBNP>3600 (median)	4.6	2.1;10.1	<0.001	5.0	1.8;14.2	0.001	2.1	0.98;4.3	0.06
Troponin T us, ng/l (median)	3.3	1.5;7.2	0.002	7.9	2.2;28.5	<0.001	2.1	0.95;4.6	0.06
Bundle branch block	2.4	0.7;7.4	0.14	1.2	0.5;3.0	0.45	1.6	0.7;3.4	0.25

Same legend as Tables 1 and 2 Supp

Table S4b: Predictors of diastolic function (Grade II+III vs I) by multivariate analyses depending on amyloidosis type patients

**Characteristics**		**AL**			**ATTRv**			**ATTRwt**	
	**OR**	**CI**	**P**	**OR**	**CI**	**P**	**OR**	**CI**	**P**
Atrial Fibrillation							0.4	0.2;0.8	0.002
NYHA class, III-IV vs I-II	5.1	2.1-12.4	<0.001	3.5	1.2;10.5	0.003			
LVEF, %	[redacted]								
IVST, mm				1.2	1.0;1.4	0.003	1.1	1.0;1.3	0.03
LVMi>170 (median)	[redacted]								
LGS, -%							0.9	0.8;0.99	0.03
NT-proBNP>3600 (median)	3.0	1.3-7.2	0.01						
Troponin T us>72ng/l (median)				4.4	1.1;17.2	0.003			

Same legend as Tables 1 and 2

Age, gender, eGFR class, SBP, bundle branch were not determinants of diastolic function in any type of CA
